# Why Drug Safety Should Not Take a Back Seat to Efficacy

**DOI:** 10.1371/journal.pmed.1001097

**Published:** 2011-09-27

**Authors:** The PLoS Medicine Editors

**Affiliations:** Public Library of Science, United Kingdom

## Abstract

The *PLoS Medicine* editors argue that methodological challenges in monitoring the safety of prescription medications should not mean that drug safety be considered less important than efficacy.

Historically, the evaluation of harmful effects resulting from prescription drug use has been considered less important than demonstrating drug efficacy, yet the harms caused by specific adverse drug reactions are a major, and avoidable, contributor to hospitalizations and deaths [1]. There are many reasons (both scientific and social) why reliable data on harmful effects may only emerge well after drug approval and marketing [2]. Some evidence suggests that drugs approved under a rapid regulatory review process may be more likely to show problems with safety post-marketing than drugs that go through a slower evaluation process [3]. And debates continue about the best ways to meaningfully synthesize and interpret data on the possible harmful effects of drugs—for example, how passive surveillance systems (spontaneous reports of suspected adverse reactions) should be improved, whether new drugs should go through a phased launch process with enhanced safety evaluations, and whether risk mitigation strategies are appropriate for drugs with safety concerns.

One such debate—whether systematic reviews estimating the risk of harmful effects should use evidence from randomized trials or observational studies—seems finally to have been laid to rest. In a systematic overview published earlier this year in *PLoS Medicine* [4], Su Golder, Yoon Loke, and Martin Bland demonstrate that, for 19 specific drug–harm relationships, the evidence on magnitude of risk for each particular harm discovered through systematic reviews of randomized trials was, on average, no different from the evidence assembled via systematic reviews of observational studies. This is an important finding, although perhaps counterintuitive: it is easy to imagine that observational studies would be so plagued by confounding that the estimates of risk of harm they generate could be biased away from true effects. The implications of this study for future evaluations of drug safety are clear: systematic reviewers should consider all types of evidence in trying to build a complete picture of harms associated with drug treatments.

In another study published this week in *PLoS Medicine* [5], Patricia McGettigan and David Henry report their re-evaluation of one specific and much-studied harmful effect—that of cardiovascular risk associated with use of nonsteroidal anti-inflammatory drugs (NSAIDs). Many previous systematic reviews have been conducted, largely using evidence from randomized trials, but these trials have generally captured only small numbers of cardiovascular events and have focused mainly on a small range of specific NSAIDs. By revisiting observational data in their systematic review, Henry and colleagues were able to form a fuller profile of the cardiovascular risks associated with use of a much wider group of NSAIDs, across dose ranges and in population settings, than had previously been the case. Broadly, their findings correlate closely with those of systematic reviews of trial data, but also show that there seems to be no “safe” lower dose for cardiovascular risk associated with certain NSAIDs, such as rofecoxib and diclofenac.

These studies together highlight the importance of data from high-quality observational studies in enabling estimation of the risk of harms associated with specific drug treatments. Passive surveillance is still crucial for providing early warning signals and generating new hypotheses about possible harms associated with specific approved drugs. However, new hypotheses emerging from such surveillance must subsequently be explicitly tested, preferably using study designs that can incorporate data on the size of the exposed population (such as cohort or record linkage studies). Such studies can therefore estimate the relative increase in risk associated with exposure, which is difficult or impossible to calculate from passive surveillance data.

A new initiative established by the European Medicines Agency (ENCEPP, the European Network of Centres for Pharmacoepidemiology and Pharmacovigilance [6],[7]) seeks to promote the conduct of such studies and establish standards for post-marketing safety evaluations. Given the diversity of designs and multiple possible sources of bias in pharmacoepidemiology, this will not be an easy job. But the initiative is already showing signs of setting high standards in some areas. Studies conducted solely by industry will not be eligible to qualify for ENCEPP approval; studies must be publicly registered before collection of data, and protocols and datasets must be released (with some restrictions relating to data privacy) in a timely way after completion. Some vague wording in the ENCEPP code of conduct remains, however: “datasets” can be interpreted to mean analyzed, not raw, data, meaning that other investigators may not be able to exploit the full potential of the data in conducting reanalyses. Critically, ENCEPP can still potentially approve studies funded by the pharmaceutical industry, with involvement of industry partners in design and analysis, providing the study's lead investigator is based within an ENCEPP-approved center. More worryingly, the code allows for industry sponsors to retain control of datasets; this, and other provisions, may enable conflicts of interest to creep in during study design or data analysis.

Clearly, for post-approval safety studies, one size will not fit all. Conduct and reporting are unlikely to be standardizable in the same way as has been possible for randomized trials, in which there is agreement on what information needs to be registered about the study and when [8], and specific standards for the reporting of studies, such as CONSORT [9], are widely accepted. The ENCEPP guidance avoids normative statements about study design, instead preferring to highlight the methodological challenges and multiple sources of bias that plague analysis and interpretation of data. However, these challenges should not discourage investigators, regulators, and patients from demanding a higher safety standard for approved drugs. Higher standards will require both greater transparency—in revealing what studies are being conducted and what data that have been generated—and greater willingness of funders to support new studies specifically addressing drug safety.

## References

[1] Pirmohamed M, James S, Meakin S, Green C, Scott AK (2004). Adverse drug reactions as cause of admission to hospital: Prospective analysis of 18,820 patients.. BMJ.

[2] Strom B (2004). Potential for conflict of interest in the evaluation of suspected adverse drug reactions.. JAMA.

[3] Carpenter D, Zucker EJ, Avorn J (2008). Drug-review deadlines and safety problems.. N Engl J Med.

[4] Golder S, Loke YK, Bland M (2011). Meta-analyses of adverse effects data derived from randomised controlled trials as compared to observational studies: Methodological overview.. PLoS Med.

[5] McGettigan P, Henry D (2011). Cardiovascular Risk with non-steroidal anti-inflammatory drugs: Systematic review of population-based controlled observational studies.. PLoS Med.

[6] European Network of Centres for Pharmacoepidemiology and Pharmacovigilance.. http://www.encepp.eu/.

[7] Schneeweiss S, Avorn J (2011). Postmarketing studies of drug safety.. BMJ.

[8] Laine C, Horton R, DeAngelis CD, Drazen JM, Frizelle FA (2007). Clinical trial registration — Looking back and moving ahead.. N Engl J Med.

[9] CONSORT: Transparent Reporting of Trials.. http://www.consort-statement.org/.

